# Computational neuroanatomy: ontology-based representation of neural components and connectivity

**DOI:** 10.1186/1471-2105-10-S2-S3

**Published:** 2009-02-05

**Authors:** Daniel L Rubin, Ion-Florin Talos, Michael Halle, Mark A Musen, Ron Kikinis

**Affiliations:** 1 Department of Radiology, Stanford University School of Medicine, Stanford, CA, USA; 2 Stanford Medical Informatics, Stanford University School of Medicine, Stanford, CA, USA; 3 Department of Radiology, Brigham and Women's Hospital, Harvard Medical School, Boston, MA, USA

## Abstract

**Background:**

A critical challenge in neuroscience is organizing, managing, and accessing the explosion in neuroscientific knowledge, particularly anatomic knowledge. We believe that explicit knowledge-based approaches to make neuroscientific knowledge computationally accessible will be helpful in tackling this challenge and will enable a variety of applications exploiting this knowledge, such as surgical planning.

**Results:**

We developed ontology-based models of neuroanatomy to enable symbolic lookup, logical inference and mathematical modeling of neural systems. We built a prototype model of the motor system that integrates descriptive anatomic and qualitative functional neuroanatomical knowledge. In addition to modeling normal neuroanatomy, our approach provides an explicit representation of abnormal neural connectivity in disease states, such as common movement disorders. The ontology-based representation encodes both structural and functional aspects of neuroanatomy. The ontology-based models can be evaluated computationally, enabling development of automated computer reasoning applications.

**Conclusion:**

Neuroanatomical knowledge can be represented in machine-accessible format using ontologies. Computational neuroanatomical approaches such as described in this work could become a key tool in translational informatics, leading to decision support applications that inform and guide surgical planning and personalized care for neurological disease in the future.

## Background

A goal for translational research in neuroscience is to understand and cure crippling neuropsychiatric diseases such as Parkinson's disease, to track chronic diseases as symptoms progress and remit; and to guide precise neurosurgical interventions while sparing the normal tissue underlying critical cognitive functions and behaviors. There is an unprecedented opportunity to understand and treat neurological diseases, given the significant progress in the variety and sophistication of neuroimaging techniques and the rapid accumulation of neuroscientific data in electronic form. Translational biomedical informatics methods are becoming critical to scientific progress by organizing and disseminating new neuroscientific knowledge in this highly complex and rapidly evolving domain.

Imaging is key aspect of the evaluation of neuropsychiatric disease. Images provide spatial information about anatomic structures in the brain that is critical to neurosurgeons in planning interventional procedures. However, the images themselves lack the anatomic and functional knowledge (excitatory and inhibitory properties of connections) that are critical in surgical planning. Integrating rich anatomic and functional knowledge with the spatial information in images is thus a critical task for clinical care and surgical planning for neurosurgical patients. The explosion in electronically accessible knowledge needed to inform these tasks is necessitating development of computational approaches to help researchers and clinicians manage and use the knowledge effectively.

**Figure 1:**
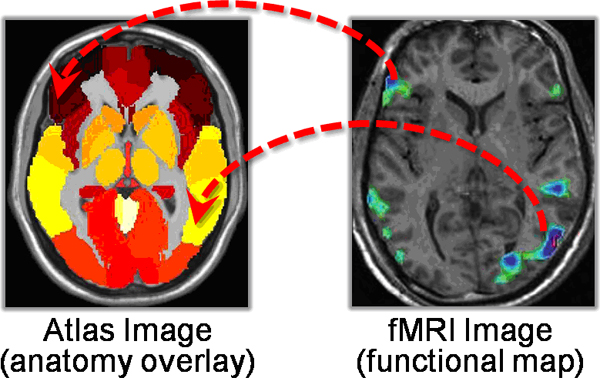
**Image atlas of the brain**. Image atlases represent spatial information by providing a parcellation of the anatomic structures contained in the brain (left). Each structure is represented as a spatial region of uniform color. Other anatomic knowledge about the structure, such as functional information, is not represented. Image atlases are generally used to infer the anatomic localization of brain structures in individual subjects by registering their images to the atlas. For example, the anatomic identity of areas of activity in fMRI are identified in this manner (right).

Image atlases are one promising computational approach for representing and characterizing neurological disease. Image atlases are representations and databases of anatomical and spatial information that capture significant attributes of the brain from imaging studies and that inform the creation of robust mathematical models [1]. They are produced by segmenting images of the brain in a single idealized subject to produce an image map, labelling each structure to identify the anatomic structures, and registering images from individual patients with the atlas to infer the location of structures (Figure 1).

While image atlases are making important contributions to neuroscience, they lack complete knowledge about their contents, such as how anatomic structures are connected, and the functional significance of abnormalities in various structures. Functional information – whether a connection is excitatory or inhibitory – is not represented. To develop applications that can help physicians to tailor optimal treatment for neurological disease, such as surgical treatment planning and personalized care, we need computational methods to integrate and access both spatial anatomic information and functional knowledge about the contents of neuroanatomical images.

Ontologies provide a means to make the anatomic and functional neuroanatomical knowledge explicit for machine processing and accessible to decision support applications. An ontology specifies the entities, their attributes and relations in a domain, providing an explicit, human-readable and machine-accessible structured description of the domain. Ontologies are being embraced in biology to express a common vocabulary, shared understanding, and complex relationships among diverse biological data in a way that is useful for both human understanding and automated computer reasoning; in fact, ontologies have opened entire new avenues for organizing, integrating and retrieving biological data [2]. We believe ontologies will be advantageous in representing the knowledge in the neurosurgical domain, and could provide the computational substrate to enable a variety of intelligent applications, such as surgical planning decision support and computerized training.

Our hypothesis is that it is possible to create an ontology-based representation of anatomic and functional neuroanatomical knowledge. The current work is an extension of our recent research [3], focusing on our methodology for ontological modeling of the neural components, connections, and enablement of computational reasoning using this model. The ontology can support automated reasoning and inform practitioners about the functional consequences of deranged neural connectivity. This functionality could ultimately be useful for automated computer reasoning tasks such as surgical planning. We undertook this work to demonstrate the feasibility of our approach in a focused use case.

Our ultimate goal is to integrate ontologically-modeled knowledge of anatomy and function with geometric brain atlas information (label maps and three-dimensional models) derived from high-resolution, multi-modal imaging. Such integrated spatial and anatomic knowledge will enable image-based reasoning applications and personalized care for individual patients.

## Methods

**Figure 2:**
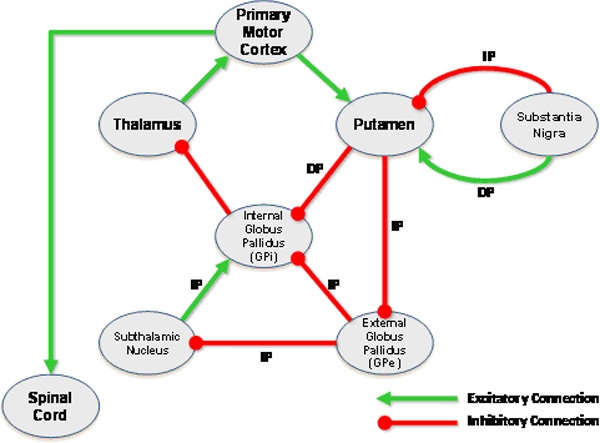
**Functional organization of the motor initiation neural network**. This figure is a diagrammatic representation of the major brain structures and connections related to the motor initiation network. Anatomically significant neural pathways are labeled ("DP" = Direct Pathway; "IP" = Indirect Pathway). Functional properties of neural connections (excitatory or inhibitory) are indicated by the color of the connections (line connectors in the diagram).

As we previously described [4], we extracted the relevant functional neuroanatomical information needed to represent the functional organization of the motor initiation neural network from authoritative neuroscience textbooks [3,5-7] The anatomic knowledge was summarized as a diagram indicating the major anatomic components and their neural connections (Figure 2). Certain neural connections have particular anatomic importance, such as whether they belonged to the direct or indirect pathway; this information was conveyed using labels on the connectors in the diagram. Finally, the neural connections have dominant functional activity in terms of being primarily excitatory or inhibitory on the nuclei to which they connect, indicated by the color and shape of the connectors in the diagram (Figure 2). The representation of excitatory and inhibitory connections in these tracts reflected the model commonly used in neurosurgical evaluation: +1 for excitation and -1 for inhibition.

**Figure 3:**
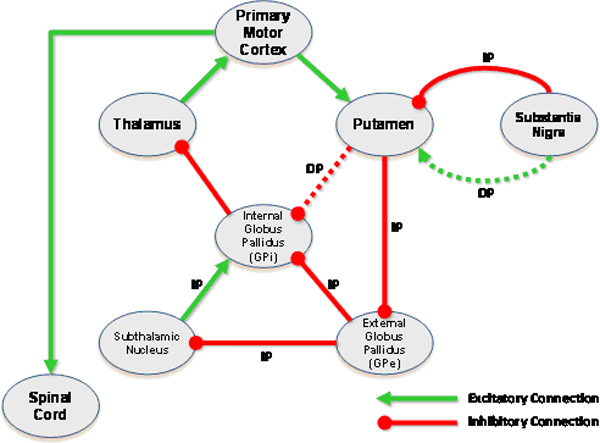
**Pathological alterations of neural circuits in disease**. This figure illustrates the abnormal connections (dashed line connectors) that exist in Parkinson's disease, characterized by impaired activity in the direct basal ganglia pathway relative to the indirect pathway. Note that this results in an imbalance of activation in the neural network.

In addition to collecting knowledge about canonical normal anatomy, we acquired knowledge about Parkinson's disease, a disorder affecting the motor initiation network. In Parkinson's disease, there is degeneration of neural elements, leading to a decrease in the activity of the direct basal ganglia pathway relative to the indirect pathway activity (Figure 3). This, in turn, results in an increased inhibitory output from the internal pallidal segment (globus pallidus pars interna, GPi resulting from unbalanced inhibition), ultimately resulting in decreased cortical stimulation and elicitation of the symptoms of the disease – a hypokinetic movement disorder characterized by impaired initiation of movement, reduced velocity and amplitude of movement, and resting tremor with increased muscle tone.

**Figure 4:**
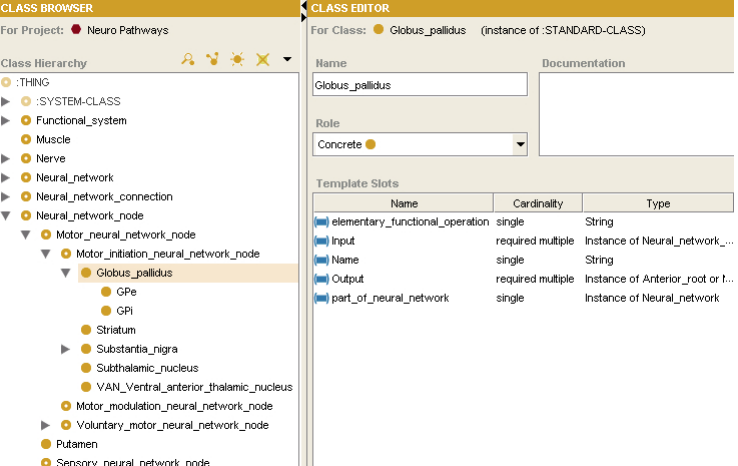
**Ontology of functional neuroanatomical knowledge**. The ontology (shown in Protege) contains classes representing anatomic components of the nervous system (nerves, nuclei, and connections) and the functional organization of the nervous system (neural networks and functional systems). Each class contains slots – attributes of the classes which provides the anatomic and functional knowledge in this representation. For example, the anatomic entity Globus Pallidus is seen to have an input and output, an elementary functional operation, and a neural network of which it is a component.

We created an ontology of functional neuroanatomy, based on the anatomic knowledge we had acquired for normal and disease states (Figure 2 and Figure 3). The ontology was built using a disciplined modeling approach, inspired by that adopted by the Foundational Model of Anatomy [8]; in fact, where possible, anatomic entities from the FMA were used in our ontology. However, FMA does not describe neural connectivity to form neural pathways, nor does it describe the functional aspects of neural connections (excitation and inhibition). Thus, we extended our ontology to include this knowledge in the form of attributed relations, similar to prior work in creating symbolic models of cardiovascular physiology [9]. The final ontology contains 235 classes, 50 slots (attributes), and 394 instances. The top-level class hierarchy of the ontology is organized into major axes of neuroanatomical information: functional system, nerve, neural network, neural network connection, and neural network node (Figure 4).

**Figure 5:**
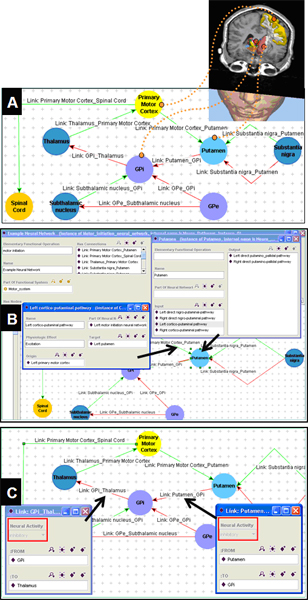
**Representing neuroanatomical knowledge in ontology**. (A) Anatomic structures in the brain are represented as instances in an ontology of neuroanatomy/connectivity and displayed as a graph, showing anatomic structures (nodes) and connections (arcs) similar to the diagrammatic representation of the same knowledge (Figure 2). (B) Nodes and arcs contain attributes making their inputs and outputs explicit. (C) The functional behavior of each connection (excitatory or inhibitory) is represented as attributes on the connectivity relations.

In our ontology, anatomic structures and connections are represented by instances of ontology classes of the corresponding anatomic entities (Figure 4). In order to visualize the neuroanatomical ontology-based model, we used the Protégé Diagram Widget,[10] which provides a graphical paradigm for creating, linking, and visualizing instances created using an ontology. We created different glyphs to represent the different types of components in neuroanatomical model to clarify the distinction among different types of anatomic structures (Figure 5). The instances contain attributes indicating how the anatomic structures are connected to other components. Other parts of the ontology specify the functional organization of the brain – which groups of nuclei and connections correspond to neural pathways. Functional information about neural connections was specified qualitatively in the ontology using a NeuralActivity attributed relation with values of "excitatory" and "inhibitory" (Figure 5C). For our representation of normal neural anatomy in the ontology, there are nine instances of excitatory neural connections and nine instances of inhibitory neural connections.

To represent abnormal connections, we altered the normal model, creating abnormal neural connections that had the appropriate values for the NeuralActivity attribute appropriate to the abnormality. Specifically, if a neural connection was impaired, then the value of NeuralActivity was set to "no activity". Alternatively, we could represent such connections by deleting the corresponding arcs in the model. For example, for Parkinson's disease, there are two instances representing impaired connections: impaired excitation from from Substantia Nigra to Putamen, and impaired inhibition from Putamen to GPi (Figure 3). In the ontology, these instances are created from the Impaired_Excitatory_Neural_Connection class, both having "no activity" for the value of the NeuralActivity attribute.

**Figure 6:**
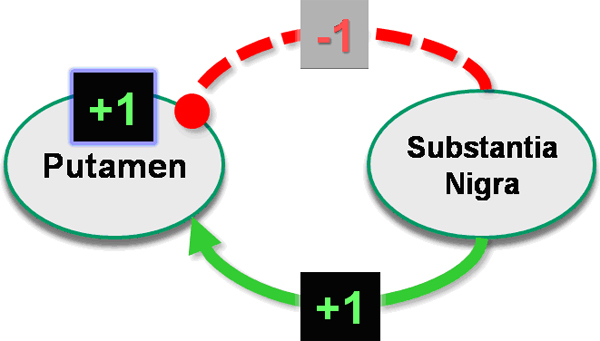
**Calculating net activation in brain nuclei**. Anatomic structures in the brain are connected to other structures via neural tracts, whose net effect on a particular brain nucleus is either excitatory or inhibitory. The net activation of a particular nucleus is calculated by summing all incoming excitatory connections and subtracting the incoming inhibitory connections, and assuming equal weight for each connection (+1 for excitation and -1 for inhibition). In this example, the tract connecting substantia nigra to putamen is deranged, and its inhibitory input to putamen is lacking. Thus, there is net activation of the putamen ("+1").

To assess the potential benefits of our approach, we interviewed a neurosurgeon with neuroanatomical expertise who evaluated our models and qualitatively compared the benefits of our explicit, structured neuroanatomical representation with the non-computational alternative (all information processing in the head of the practitioner). In addition, we explored the possibility of creating an automated reasoning application by manually applying an algorithm to calculate net activation of particular nuclei in neural network models. Net activation was calculated by visiting each node in the model and summing all incoming excitatory connections, while subtracting the sum of all incoming inhibitory connections. For this calculation, excitation and inhibition were equally weighted (+1 for excitation and -1 for inhibition). The net value produced in this manner determined whether each nucleus was excited or inhibited, and subsequently propagated to downstream nuclei in the network (Figure 6).

We applied this algorithm to calculate the net activation of motor cortex (the ultimate target of interest in diseases of the motor initiation network). This was performed by iterating all neural connections, commencing with nuclei receiving no inputs, and calculating the net activation at each brain nucleus, propagating the net activation of each nucleus along the neural pathways until the cortex was reached. We compared the results in the normal state model and in the Parkinson's disease state model.

## Results

We used our ontology to build a representation of the normal motor initiation network by creating ontology instances for the anatomic entities to which they correspond. Accordingly, there is a one-to-one mapping from each instance in the ontology (each node in the graphical view of the ontology) to each structure in the brain (Figure 5). This correspondence provides the link between spatial image information and ontology-based anatomic knowledge.

The ontology model also represented the connections among brain components (arcs in the graphical view), each having the appropriate attributes to specify the functional connectivity (excitatory or inhibitory; Figure 5). For example, the Putamen is represented as an instance of NeuralNetworkNode (Figure 5B). Connections between brain regions were represented by creating instances of the NeuralConnection class. Accordingly, the connection between the Putamen and the primary motor cortex was established by creating the arc, LinkPrimaryMotorCortex_Putamen. The arcs (neural connections) were assigned the necessary value for the NeuralActivity attribute (excitatory or inhibitory) as required to model the functional neuroanatomic knowledge (Figure 5C). The resulting graphical model of brain anatomy in the ontology representation had a very similar appearance to the diagrammatic representation from the knowledge source used to create the model (compare Figure 2 and Figure 5).

**Figure 7:**
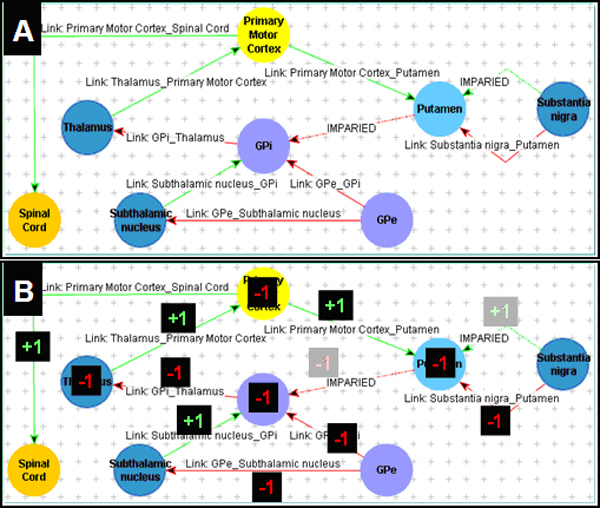
**Representing neuroanatomical abnormalities**. Abnormal anatomic structures are represented by altering the attributes of their connections to other structures. (A) Motor initiation neural network in Parkinson's disease, showing impaired connections (decrease in activity) in the direct basal ganglia pathway relative to the indirect pathway activity (dotted arc, labeled "IMPAIRED") as well as impaired putamino-pallidal connections. (B) Net activation of brain structures can be computed in the ontological representation of the neuroanatomical network by propagating net activation at each nucleus. In this representation of the Parkinson's disease, each connection is assumed to have unitary activation or inhibition, and there is net inhibition of the cortex.

Our ontology could also represent abnormalities in disease states with abnormal functional connectivity. Specifically, we represented the neural network in Parkinson's disease (Figure 3). As with the model for the normal state for the motor initiation network, the graphical model of the ontological representation of functional neuroanatomy in the disease model had a very similar appearance to the diagrammatic representation from the knowledge source used to create this model (compare Figure 3 and Figure 7A).

In addition to providing a structured representation of the information needed to create a graphical symbolic display of the neuroanatomical knowledge, the ontology provided a computational infrastructure to evaluate the functional consequences of connectivity derangements in the neural networks we studied. In the Parkinson's model, we could evaluate the net activation in different brain nuclei as a consequence of the functional derangements in the connections affected in this disease. For example, by tracing the connections from the SubstantiaNigra to the PrimaryMotorCortex, we could conclude that there is net inhibition of the latter (Figure 7B). While in our particular model both excitation and inhibition were equally weighted according to the neurosurgical perspective, the knowledge representation and processing algorithm could be altered to reflect alternative functional neuroanatomic knowledge, such as real-valued excitation and inhibition.

Similarly, we could use this model as a platform to infer the consequences (in terms of net activation) resulting from different surgical interventions that would disrupt particular neural connection pathways. Such inference could be useful in guiding surgical planning. In combination with the schematic view of the ontology-based model, users could interrogate particular portions of the model to study the functional aspects of neural connectivity. The neurosurgical domain expert who had developed the neural network models by hand believed that our computational approach would be beneficial for simulation and surgical planning in complex cases.

## Discussion

Computational methods can transform rapidly accumulating biomedical data into proactive, predictive, and participatory health solutions. Ontologies are a key tool in the translational bioinformatics arsenal, because they provide explicit, machine-processable and human-comprehensible descriptions of biomedical data elements and entities needed for computers to help people make sense of the wealth of biomedical information. Neuroscience is a complex domain, rich in anatomic and functional knowledge. Our goal was to develop an ontology-based symbolic model of structural and functional neuroanatomy. The ultimate objective is to use this computationally accessible knowledge to drive a decision support system for surgical planning in a variety of neurological diseases.

In the current study, we have demonstrated the feasibility of encoding the knowledge necessary to describe the basic functional organization of the motor system. We chose the motor system because it displays little anatomic variability and its function is better characterized than that of other, more complex functional systems in the brain. Furthermore, the motor system is involved in several important pathologic processes with high impact on public health, such as movement disorders. Although the scope of our current prototype ontology is limited to a single functional system, we believe that our methods are extensible, and that our modeling approach will be applicable to a richer breadth of neural networks. For example, we have already demonstrated that a common ontological framework can describe both normal as well as pathological neurological states (Figures 2, 3, 5 and 7).

Our ontology encodes two complementary aspects of neuroanatomy: (a) a structural aspect, concerned with spatially-localized structures and relationships, and (b) a functional aspect, dealing with physiological aspects of neural connections between neural structures – excitation or inhibition of one nucleus on another exerted via neural pathways. The structural knowledge is represented in the topology of the network of ontological components (Figure 5B), while the functional aspects are represented as attributes on the connections between neural components (Figure 5C). The ontology provides a facile mechanism for neuroscience practitioners to browse and edit the neuroanatomical knowledge, because it can be displayed in a graphical form similar to that which they are accustomed (compare Figure 2 and Figure 5).

Our methods are a direct extension of previous endeavors we undertook for creating explicit and computable representations of hemodynamic models of the cardiovascular system [9]. In that work, we also created an ontology-based model of structural and functional components of a biomedical system, albeit in the cardiovascular domain. One can view the neuroanatomical models in the current work as completely analogous, comprising both structural and functional components whose attributes are specified explicitly in the ontology.

There are several limitations of our work. Our current representation assumes a simple ternary-valued activation of connections – excitatory, inhibitory, or deranged (no activity). In reality, such connections are likely real-valued, as neural tracts comprise many fibers, some of which are activated and some not. Our choice was guided by the neurosurgical perspective that makes this simplifying assumption; certainly our representation could be altered to accommodate real values for activation in the future.

Another limitation is that we have represented only the motor initiation network of the brain, and we have not represented the full spectrum of neural diseases. We believe our modelling approach is extensible to other neural systems beyond the motor network, given that such systems comprise nuclei and connections. The value of our methods in modelling other diseases would need to be studied, however. In addition, we believe computational approaches to neuroanatomy such as described in this report will be useful mainly in complex conditions – an expert would not likely need assistance in simple or well-known scenarios. However, as the richness in anatomic and functional neural knowledge expands, we believe our framework will be useful to organizing this knowledge and making it computationally accessible to applications.

There are benefits to our approach. First, we have made neuroanatomic knowledge explicit in the ontology, in a format that is both human-readable (in the Protege Diagram Widget) and machine-accessible. The ontological representation can be modified directly in the diagram, and functional consequents can be immediately deduced from the ontology.

Another benefit of our approach is that the neuroanatomical knowledge is in a machine-accessible format. Computer reasoning applications can be created to process the anatomic knowledge in intelligent ways, such as in surgical planning applications capable of identifying optimum targets for functional stereotactic surgery. A decision support application informed by the richness of structural and functional anatomic knowledge could also guide the decisions about the optimum tissue ablation path in such surgical interventions. We have already shown here that the functional consequences in a disease state can be inferred by traversing the connections in the model and calculating the net activation of different brain regions (Figure 6 and Figure 7). We are currently creating an application to derive these inferences automatically, by propagating activation across the neural network, informing practitioners about the functional consequences of neural connectivity derangements and interventions.

A potential application for our work is an ontology-augmented neuroanatomy atlas, serving as the basis for a multitude of intelligent applications that can combine processing of spatial information with analysis of the function of the corresponding regions in images. Such knowledge-enhanced atlases can enable applications for simulation and neuroanatomy teaching. Symbolic models of functional neuroanatomy, alone or in combination with digital brain atlases, could pave the way for future knowledge-based applications for neuroscientific research and clinical care.

## Conclusion

We have shown that functional neuroanatomical knowledge can be represented in a computational format using an ontology. The ontology provides a means to peruse the knowledge, while making it accessible to computer reasoning, such as decision support, modeling, and teaching applications.

## Competing interests

The authors declare that they have no competing interests.

## Authors' contributions

DLR and IFT conceived this work, acquired the anatomic knowledge, and produced the computational models in this work. MH developed code to enable access to the anatomic knowledge. MAM and RK participated in its design and coordination of this study. All authors read and approved the final manuscript.
